# Dietary 1.3-1.6 yeast β-glucans enhance immune response and disease resilience in European seabass challenged with *Tenacibaculum maritimum*

**DOI:** 10.3389/fimmu.2026.1798375

**Published:** 2026-04-20

**Authors:** Miguel Cabano, Nadège Richard, Julie Schulthess, Lourenço Ramos-Pinto, Francisco Pinto-Cunha, Maria João Peixoto, Cláudia Aragão, Benjamin Costas

**Affiliations:** 1 Centro Interdisciplinar de Investigação Marinha e Ambiental (CIIMAR/CIMAR LA), Universidade do Porto, Matosinhos, Portugal; 2 Centro de Ciências do Mar do Algarve (CCMAR/CIMAR LA), Faro, Portugal; 3 Universidade do Algarve, Faro, Portugal; 4 Phileo by Lesaffre, Marcq-en-Baroeul, France; 5 Instituto de Ciências Biomédicas Abel Salazar, Universidade do Porto, Porto, Portugal

**Keywords:** aquaculture, bacterial pathogen, Dicentrarchus labrax, feed additives, immunization, immunonutrition

## Abstract

**Introduction:**

Nutritional strategies have emerged as promising tools to modulate immune responses in marine fish, particularly at mucosal surfaces involved in host-pathogen interactions. Dietary immunomodulators such as yeast-derived β-glucans (BG) have gained attention for their capacity to enhance innate immune defenses, however their role in coordinating these mechanisms during bacterial infections remains incompletely understood.

**Methods:**

This study evaluated the effects of two dietary inclusion levels (0.06 and 0.12%) of purified yeast β-1,3/1,6-glucans on the immune responses of European seabass (*Dicentrarchus labrax*) following a bath challenge with *Tenacibaculum maritimum* specifically. After four weeks of feeding with experimental diets, fish were exposed to a sub-lethal infection, and immune parameters were accessed in the skin, intestinal mucosa and at systemic level.

**Results:**

Growth performance and feed efficiency were not affected by BG supplementation. At 24 h post-infection, fish fed with the 0.12% BG diet maintained circulating leukocyte counts and displayed increased plasma lysozyme activity, indicating enhanced early immune readiness. Moreover skin immune responses showed an early upregulation of pro-inflammatory and antimicrobial related genes, followed by increased expression of regulatory cytokines at 7 days post-infection, suggesting an efficient transition from immune activation to resolution. In contrast, intestinal immune responses remained largely unchanged, indicating a compartmentalized modulation focused on the primary site of infection.

**Conclusion:**

Overall, dietary BG supplementation exerted a dose dependent immunostimulatory effect in European seabass, with 0.12% BG eliciting the most significant chances in disease resilience.

## 1 Introduction

Aquaculture is one of the fastest-growing food-producing sectors worldwide and plays a central role in meeting the increasing demand for high-quality aquatic animal protein. However, its expansion is constrained by infectious diseases, which compromise fish health and can cause substantial economic losses (1, 2). In addition, the intensive nature of modern aquaculture systems and the increasing restrictions on the use of antibiotics during fish farming highlight the urgent need for sustainable alternatives to maintain productivity and fish welfare (3, 4). Preventive approaches are therefore essential, particularly those that strengthen host defenses at mucosal and systemic levels. In fish, surfaces such as the skin, gills and intestine represent the first line of defense against pathogens and contain diverse immune components that contribute to pathogen recognition and elimination (5–7).

The relevance of such approaches is highlighted by *Tenacibaculum maritimum*, a Gram-negative bacterium and the etiological agent of tenacibaculosis, which remains one of the most concerning health threats to marine aquaculture. This opportunistic pathogen colonizes external surfaces such as skin, fins, gills, where it induces characteristic ulcerative lesions (8), but has also been detected in internal tissues such as the intestine (9). Infection can result in high morbidity and mortality across a wide range of species, including Atlantic salmon (*Salmo salar*) and other farmed salmonids, in which tenacibaculosis has emerged as a recurrent and economically relevant disease, as well as European seabass (*Dicentrarchus. labrax*), Senegalese sole (*Solea senegalensis*), gilthead seabream (*Sparus aurata*) and turbot (*Scophthalmus maximus*), demonstrating its broad host range and global distribution (8–11). Outbreaks are often associated with handling and environmental stressors that compromise epithelial integrity and favor infection development (12, 13). Despite advances in diagnostics and serotyping (14, 15), effective vaccines or targeted therapies are still lacking, and prophylaxis remains largely dependent on husbandry practices, emphasizing the need for complementary strategies to mitigate disease impact (1, 2).

With limited therapeutic and immunization options available, functional feeds have emerged as promising tools to improve fish health in aquaculture. Over time, the concept of functional feeds has evolved from formulations primarily focused on growth performance to diets specifically designed to support immune competence and resilience under farming conditions. These feeds typically incorporate bioactive ingredients such as probiotics, nucleotides, plant extracts, and polysaccharides, which modulate host physiology and immunity in addition to sustaining normal growth and overall performance. Among dietary immunomodulators, β-glucans have received particular attention in recent years, but their biological activity can vary substantially depending on their structural features and origin (16). β-glucans differ in linkage pattern (β-1,3; β-1,6; β-1,4), branching degree, solubility and molecular weight, and these characteristics strongly influence receptor affinity and downstream immune activation. For example, yeast-derived β-1,3/1,6 glucans (BG) remain the most widely used in aquaculture due to their strong ability to activate innate immune cells, whereas laminarin (β-1,3/1,6 glucan from brown algae) and chrysolaminarin (β-1,3 glucan from microalgae) display distinct physicochemical and immunomodulatory profiles (17, 18). Despite these structural differences, BG from multiple sources consistently stimulates key innate immune pathways, including phagocytosis, oxidative activity and cytokine secretion. Beyond their short-term immunostimulant effects, BG have been shown to induce long-lasting cellular reprogramming in teleost fish, leading to faster and more efficient responses upon secondary challenge, a phenomenon analogous to trained immunity in mammals. However, despite growing experimental evidence, this concept remains relatively underexplored and only limitedly applied in a commercial aquaculture setting, highlighting its potential as an innovative strategy to improve fish robustness under farming conditions. This trained immunity involves epigenetic and metabolic reprogramming of innate cells such as macrophages and neutrophils, resulting in an enhanced state of readiness (19). Such priming seems to be particularly relevant for fastidious pathogens like *T. maritimum*, where early activation and coordination of immune defenses are critical for survival.

Although dietary BG have been widely investigated as immunostimulants in aquaculture, their capacity to modulate mucosal and systemic immune compartments during *T. maritimum* infection remains insufficiently characterized in European seabass. In particular, whether BG can promote coordinated, tissue-specific immune responses under ulcerative bacterial challenge has not been clearly established. Given that *T. maritimum* primarily targets epithelial surfaces, understanding how dietary glucans influence mucosal immune activation alongside systemic responses is especially relevant under farming relevant infection scenarios.

To achieve this, the present study aimed to explore the immunomodulatory effects of BG in diets for European seabass following bath infection with the ulcerative Gram-negative bacterium *T. maritimum*. Both mucosal and systemic immune responses were assessed through hematological and immune parameters as well as gene expression in target tissues. In this context, specific immunoglobulin M against *T. maritimum* antigens were also analyzed after infection to gain further insights into the potential effects of BG on acquired immunity. This approach represents a first step toward further exploring the synergistic effects between immunization and tailored nutrition.

## 2 Material and methods

### 2.1 Ethics statement

All experimental procedures complied with European and Portuguese legislation on the protection of animals used for scientific purposes, specifically Directive 2010/63/EU of the European Parliament and Council and Decreto-Lei n° 113/2013 of August 7. The experiments were conducted under the supervision of trained personnel in accordance with FELASA Category C guidelines.

### 2.2 Experimental diets

Three isonitrogenous (50%), isolipidic (16%) and isoenergetic (22 MJ kg^-1^ feed) diets were formulated: a control diet was specifically formulated to meet the nutrient requirements of European seabass (Control), and two experimental diets based on the Control diet, supplemented with purified BG (> 50% purity) at 0.06 and 0.12% at the expenses of wheat meal, thereafter named as BG0.06 and BG0.12, respectively. The experimental diets were manufactured by SPAROS Lda (Olhão, Portugal). Main ingredients were ground (below 250 µm) in a micropulverizer hammer mill (SH1; Hosokawa Micron, B.V., Doetinchem, The Netherlands). Powder ingredients and oils were then mixed according to the target formulation in a paddle mixer (RM90; Mainca, S.L., Granollers, Spain). All diets were manufactured by temperature-controlled extrusion (pellet size: 1.5 mm) by means of a low-shear extruder (P55; Italplast, S.r.L., Parma, Italy). Upon extrusion, all feed batches were dried in a convection oven (OP 750-UF; LTE Scientifics, Oldham, UK) for 4 h at 45 °C. Proximate composition analyses were conducted by the following methods: dry matter, by drying at 105 °C for 24 h; ash, by combustion at 550 °C for 12 h; crude protein (N × 6.25), by high−temperature combustion (Dumas principle) with gas separation and thermal conductivity detection (LECO CN828); fat, after petroleum ether extraction, by the Soxhlet method; gross energy, in an adiabatic bomb calorimeter (IKA). Formulation and proximate composition of experimental diets are presented in **Table 1**.

**Table 1:** Formulation and proximate composition of experimental diets.

Ingredient (%)	Control	BG0.06	BG0.12
Fishmeal Super Prime ^a^	15.00	15.00	15.00
Fish protein hydrolysate ^b^	3.00	3.00	3.00
Poultry meal ^c^	5.00	5.00	5.00
Soy protein concentrate ^d^	8.50	8.50	8.50
Wheat gluten ^e^	8.40	8.40	8.40
Corn gluten meal ^f^	10.00	10.00	10.00
Guar meal ^g^	5.00	5.00	5.00
Soybean meal Hipro ^h^	14.00	14.00	14.00
Sunflower meal ^i^	5.40	5.40	5.40
Wheat meal ^j^	9.20	9.14	9.08
Vitamin and mineral premix ^k^	1.00	1.00	1.00
Choline chloride 50 ^l^	0.20	0.20	0.20
Monocalcium phosphate ^m^	1.00	1.00	1.00
L-Lysine ^n^	0.20	0.20	0.20
Yeast β-1,3/1,6 glucan (BG) °	0.00	0.06	0.12
Fish oil ^p^	5.00	5.00	5.00
Salmon oil ^q^	5.00	5.00	5.00
Rapeseed oil ^r^	4.10	4.10	4.10
Proximate composition (% as fed)			
Dry matter	89.76	89.85	89.99
Ash	6.50	6.50	6.48
Crude protein	49.32	49.53	49.75
Crude fat	16.47	15.98	16.05
Gross energy (MJ kg^-1^)	22.18	22.68	22.14

a Fishmeal Super Prime: 66.3% crude protein (CP), 11.5% crude fat (CF), Pesquera Diamante, Peru.

b CPSP90: 82.6% CP, 9.6% CF, Sopropêche, France.

c Poultry meal: 62.4% CP, 12.5% CF, SAVINOR UTS, Portugal.

d Soycomil P: 62.2% CP, 0.87 CF, ADM, The Netherlands.

e VITAL: 80.4% CP, 5.8% CF, ROQUETTE Frères, France.

f Corn gluten meal: 61.2% CP, 5.2% CF, COPAM, Portugal.

g Guar meal: 55.3% CP, 7.8% CF, Korma, Meelunie B.V., The Netherlands.

h Alphasoy 530, dehulled solvent extracted soybean meal, HiPro: 52.9 CP, 2.6 CF, ABNeo AS, Denmark.

i Dehulled solvent extracted sunflower meal, HiPro: 42.9% CP, 3.8% CF, AGP Slovakia, s.r.o, Slovakia.

j Wheat meal: 11.7% CP; 1.6% CF, Molisur, Spain.

k PREMIX Lda, Portugal: Vitamins (IU or mg kg^-1^ diet): DL-alpha tocopherol acetate, 100 mg; sodium menadione bisulfate, 25 mg; retinyl acetate, 20000 IU; DL-cholecalciferol, 2000 IU; thiamine, 30 mg; riboflavin, 30 mg; pyridoxine, 20 mg; cyanocobalamin, 0.1 mg; nicotinic acid, 200 mg; folic acid, 15 mg; ascorbic acid, 1000 mg; inositol, 500 mg; biotin, 3 mg; calcium pantothenate, 100 mg; choline chloride, 1000 mg, betaine, 500 mg. Minerals (g or mg kg^-1^ diet): cobalt carbonate, 0.65 mg; copper sulfate, 9 mg; ferric sulfate, 6 mg; potassium iodide, 0.5 mg; manganese oxide, 9.6 mg; sodium selenite, 0.01 mg; zinc sulfate,7.5 mg; sodium chloride, 400 mg; calcium carbonate, 1.86 g; excipient wheat middlings.

l Choline chloride 50%: ORFFA, The Netherlands.

m MCP: 22.7% phosphorus, 17.5% calcium, ALIPHOS, Belgium.

n L-Lysine HCl 99%, Ajinomoto EUROLYSINE S.A.S., France.

°Purified yeast fraction rich in β-1,3/1,6 glucans: 60% β-1,3/1,6 glucans; 5-10% glycogen, 5% CP; 10% CF; 10% ash; <3% mannan-oligosaccharides, Phileo by Lesaffre, France.

p Fish oil: 98.1 CF, 16% EPA, 12% DHA, Sopropêche, France.

q Salmon oil: 98.3% CF, 4.6% EPA; 5.2% DHA, Sopropêche France.

r Rapeseed oil: 98.2% CF, JC Coimbra, Portugal.

### 2.3 Experimental design

**Figure 1:**
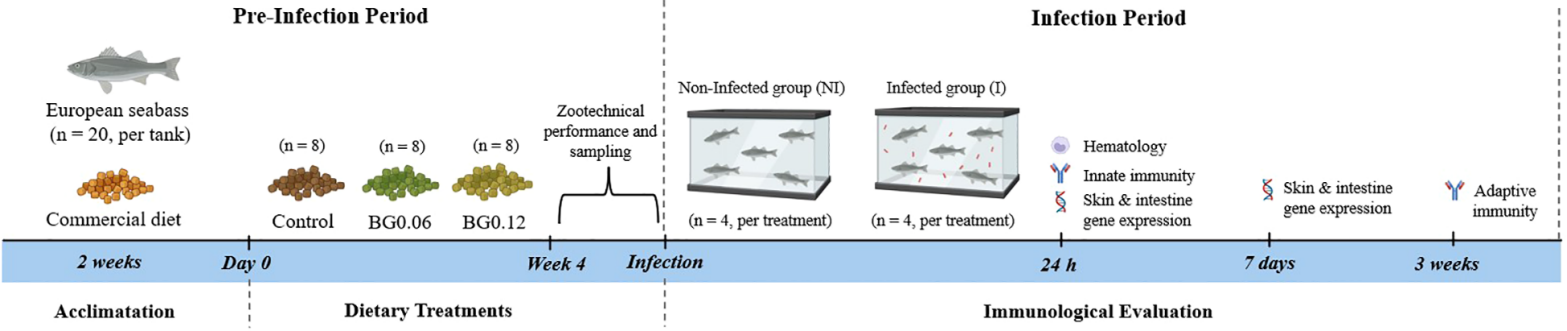
Overview of the experimental design. After a 2-week acclimatation period, European seabass were fed three diets (Control, BG0.06, BG0.12) for 4 weeks before a bath challenge with *T. maritimum* (4 × 10^5^ CFU mL^-1^). Fish were sampled at 24 h and 7 days post-infection to access hematology, innate immune responses and gene expression in skin and posterior intestine. Plasma was collected at 3 weeks post-infection to determine total and specific IgM.

The experimental design of the trial is summarized in **Figure 1**. European seabass juveniles were obtained from a commercial fish farm (ATLANTIKFISH, Portugal) and transported to the Interdisciplinary Centre of Marine and Environmental Research facilities (CIIMAR, Matosinhos, Portugal). Upon arrival, fish passed through a quarantine of 2 weeks in a 2,000 L tank at 20 °C and fed a commercial diet twice daily to ensure the absence of any signs of disease. After this period, a total of 480 fish (11.32 ± 1.01 g; mean ± SD) were individually weighed and distributed in 50 L tanks (20 fish per tank at 4.53 kg m^-3^) in a recirculating aquaculture system (RAS). Fish were assigned randomly to the three treatments, with eight replicate tanks per diet. All tanks were located in the same experimental room and supplied with filtered and heated seawater (20.26 ± 0.15 °C; mean ± SD). Water quality parameters (oxygen concentration, nitrogenous compounds, pH and salinity) were monitored daily and maintained at levels within the recommended limits for the species. All fish were submitted to a 12 h light: 12 h dark artificial photoperiod. Fish were hand fed daily at a fixed feeding rate of 1.5% of the initial tank biomass per day throughout the 4-week feeding period. After 4 weeks of feeding, fish were bulk weighed for analysis of key performance indicators (i.e., final weight, weight gain and feed conversion ratio). Following this feeding period, the experimental room was divided into two independent sections, each containing four replicate tanks per dietary treatment. One section was designated for bacterial bath inoculation (*i.e.*, infected - I), while the other section was used as a non-infected control (NI), with fish being subjected to the same handling procedures but not exposed to bacteria.

For the bacterial challenge, *T. maritimum* (strain ACC 13.1) previously isolated from Senegalese sole was used. Frozen stocks were recovered on marine agar at 25 °C for 48 h, following the procedure described by Ferreira et al. (20). Exponentially growing bacteria were collected by centrifugation (4000 × *g* for 10 min at 4 °C) and re-suspended in sterile marine broth to a concentration of 2.24 × 10^9^ CFU mL^-1^. Bacterial concentration was adjusted with the predetermined growth curve for this specific strain: y = 2 × 10^8^x + 4 × 10^7^ (21). For the infection procedure, the tank water level was reduced to 10 L, and a bacterial dosage (4 × 10^5^ CFU mL^-1^; I group) or marine broth alone (NI group) were introduced into the tanks for 2 h. This sub-lethal dosage was selected according to the outcomes of pre-challenges previously performed on the same batch of European seabass used in the present study. After this period, water inside the tanks was completely replaced to eliminate any possible remaining bacteria in the system, and thereafter water temperature was steadily increased by 5 °C to mimic the rise of temperature that triggers piscine outbreaks in aquaculture systems. Fish were monitored daily, with observations conducted twice a day (morning and afternoon) over 3 weeks.

For sampling purposes, four fish per tank were collected at 24 h and 7 days post-infection from both I and NI groups. Plasma, skin and posterior intestine samples were collected at 24 h, whereas skin and posterior intestine were sampled at 7 days post-infection. Additionally, at 3 weeks post-infection, blood was collected from all fish to obtain plasma for the analysis of total and specific IgM.

For sample collection and processing, fish were anaesthetized by immersion in 2-phenoxyethanol (500 ppm; Sigma) and sampled. Blood was collected from the caudal vein using heparinized syringes. Plasma was obtained by centrifugation (12,000 × g for 10 min at 4 °C), snap frozen in liquid nitrogen and stored at -80 °C until further analysis. Following blood collection, fish were euthanized in 2-phenoxyethanol (1,500 ppm; Sigma) and the target tissues were aseptically dissected, immediately put in tubes containing RNA later (Thermo Fisher Scientific, Massachusetts, USA), kept at 4 °C for 24 h, and stored at -80 °C after this period until RNA extraction. Total RNA was extracted using an automated extraction system (Maxwell^®^, Promega, Wisconsin, USA) according to the manufacturer’s instructions.

### 2.4 Key performance indicators

Key performance indicators were calculated using the following formulas:

Weight gain (%) = 100 × (final body weight − initial body weight) × initial body weight^-1^.

Feed conversion ratio (FCR) = apparent feed intake × (final body weight − initial body weight)^-1^.

Survival = 100 × (final number of fish x initial number of fish^-1^).

### 2.5 Analytical procedures

#### 2.5.1 Hematological parameters

The hematological profile was assessed following Machado et al. (22). Total white (WBC) and red (RBC) blood cell counts were performed using a Neubauer chamber, and hemoglobin concentration was measured with a commercial kit (SPINREACT, ref. 1001230, Spain) (n = 12 per diet × infection condition).

#### 2.5.2 Assessment of humoral immune responses

Plasma lysozyme activity (n = 16 per diet × infection condition) was measured following Costas et al. (23), through the EnzChek Lysozyme Assay Kit (E-22013) manufacturer’s protocol with minor modifications. Briefly, plasma samples were diluted 1:5 in reaction buffer (0.1 M sodium phosphate, 0.1 M NaCl, pH 7.5, with 2 mM sodium azide). A six-point standard curve (500–31.25 U mL^-1^) was prepared using serial dilutions of a 1000 U mL^-1^ lysozyme stock solution. Samples and standards (50 μL per well) were loaded in duplicate into a black 96-well fluorescence plate, followed by the addition of 50 μL of DQ lysozyme substrate (50 μg mL^-1^), a fluorescein-conjugated *Micrococcus lysodeikticus* suspension. Fluorescence was recorded every 5 min for 30 min at 37.5 °C using an excitation/emission of 494/518 nm. The blank (no enzyme control) was subtracted from all readings, and lysozyme activity (U mL^-1^) was calculated based on the standard curve.

Total plasma IgM levels (n = 16 fish per diet × infection condition) were quantified by ELISA following Cuesta et al. (24), with slight modifications. Flat-bottomed 96-well plates were coated with plasma samples diluted 1:100 in 50 mM Na_2_CO_3_ buffer (pH 9.6) (100 µL per well) and incubated for 1 h at room temperature (RT). Plates were then washed three times with T-TBS (20 mM Tris-HCl, 150 mM NaCl, 0.05% Tween-20, pH 7.5) and blocked with 5% (w/v) low-fat milk in T-TBS for 1 h at RT. After washing with T-TBS, plates were incubated for 1 h at RT with 100 µL per well of monoclonal anti-European seabass IgM antibody (1:100 in blocking buffer; Aquatic Diagnostics Ltd.). Plates were washed again with T-TBS and incubated with a secondary anti-mouse IgG-HRP conjugate (1:1000 in blocking buffer) for 1 h at RT. The reaction was developed with 100 µL TMB per well for 5 min, stopped with 100 µL H_2_;SO_4_; (2 M), and the absorbance was read at 450 nm using a Synergy HT microplate reader (BioTek Instruments).

*T. maritimum*-specific plasma IgM levels (n = 16 fish per diet × infection condition) were quantified by ELISA following Raida et al. (25), with slight modifications. High-binding flat-bottomed 96-well plates were coated with 100 µL per well of sonicated *T. maritimum* antigen (5 µg mL^-1^) prepared in 50 mM Na_2_CO_3_ buffer (pH 9.6) and were left for incubation overnight at 4 °C. Plates were washed three times with T-TBS and blocked with 5% (w/v) low-fat milk in T-TBS overnight at 4 °C. After blocking, plates were washed three times with T-TBS and incubated with plasma samples diluted 1:100 in 50 mM Na_2_CO_3_ buffer (pH 9.6) and incubated overnight at 4 °C. Plates were then blocked with 5% (w/v) low-fat milk in T-TBS for 1 hour at 4 °C. After this step, plates were then washed three times with T-TBS and incubated for 1 h at RT with 100 µL per well of monoclonal anti-European seabass IgM antibody (1:100 in blocking buffer; Aquatic Diagnostics Ltd.). After washing with T-TBS, a secondary anti-mouse IgG-HRP conjugate (1:1000 in blocking buffer) was added and incubated for 1 h at RT. The reaction was developed with 100 µL TMB per well for 5 min, stopped with 100 µL H_2_SO_4_ (2 M), and absorbance was read at 450 nm using a Synergy HT microplate reader (BioTek Instruments).

Antiprotease activity (n = 12, per diet × infection condition) was measured as described in Ellis (1990) (26) with some modifications. Briefly, 10 µl of plasma was incubated in duplicates with an equal volume of trypsin solution (5 mg mL^-1^; in NaHCO_3_, pH 8.3) for 10 min at room temperature. The reaction mixture was then supplemented with phosphate buffer and azocasein solution and further incubated for 1 h at room temperature. The reaction was stopped by adding 250 µl of trichloroacetic acid, followed by centrifugation (10000 × g, 5 min, room temperature). Next 100 µl of the supernatants were transferred to a 96-well plate already containing 100 µl NaOH in each well, and absorbance was measured at 450 nm. Phosphate buffer blanks and reference samples were used to calculate the percentage inhibition of trypsin activity.

Total peroxidase activity (n = 12, per diet × infection condition) in plasma was assessed following the method described by Quade and Roth (27), with minor modifications. Briefly, 15 µL of intestinal homogenate were diluted in 135 µL of HBSS (without Ca^2+^ and Mg^2+^) and added in triplicate to a flat-bottomed 96-well plate. Subsequently, 50 µL of 20 mM 3,3’,5,5’-tetramethylbenzidine (TMB; Sigma) and 50 µL of 5 mM H_2_O_2_ were added. After 2 minutes of incubation at room temperature, the reaction was stopped with 50 µL of 2 M H_2_SO_4_. Absorbance was measured at 450 nm using a Synergy HT microplate reader. Wells without sample were used as blanks. One unit of peroxidase activity was defined as the amount of enzyme required to produce a change of 1 OD unit.

#### 2.5.3 Gene expression

Total RNA was extracted from posterior gut and skin tissues (n = 16, per diet × infection condition) using the Maxwell^®^ RSC Instrument (Promega, USA) and the Maxwell^®^ RSC simplyRNA Tissue Kit, following the manufacturer’s instructions. Tissue homogenization was performed using a Precellys homogenizer to ensure efficient cell disruption. RNA concentration and purity were assessed with a DS-11 Spectrophotometer (DeNovix, USA), and RNA integrity was evaluated by agarose gel electrophoresis to ensure that degradation was not present in samples, comparing RNA bands to a molecular marker. Samples were treated with DNase I (GRiSP Research Solutions, Porto, Portugal) to eliminate genomic DNA contamination. First-strand cDNA was synthesized from 1 μg of total RNA using the NZY First-Strand cDNA Synthesis Kit (NZYTech, Lisbon, Portugal), following the manufacturer’s protocol. Real-time quantitative PCR (qPCR) was performed on a CFX384 Touch Real-Time PCR Detection System (Bio-Rad, USA). Each 10 μL reaction contained 1 μL of diluted cDNA (1:4), 5 μL of NZYSpeedy qPCR Green Master Mix (NZYTech, Portugal), 3.2 μL of nuclease-free water, and 0.4 μL (10 μM) of each gene-specific primer. Amplification was carried out with primers targeting genes involved in the immune response. Accession numbers, annealing temperatures, efficiency values, and primer sequences are listed in **Table 2**. Primers sequences were identified by searching in database (NCBI, database v1.0c seabass genome) and were designed using NCBI Primer-BLAST and IDT OligoAnalyzer ToolTM, following standard qPCR design criteria such as amplicon size, Tm compatibility, GC content, and avoidance of self- or cross-dimer formation. The efficiency of primer pairs was evaluated using a standard curve based on serial two-fold dilutions of cDNA, calculated from the slope of the regression line of cycle threshold (Ct) values versus relative cDNA concentration (28). Melting curve analysis was performed to confirm amplification specificity and the absence of primer dimers. The standard cycling conditions were as follows: initial denaturation at 95 °C for 10 min; 40 cycles of 95 °C for 15 s followed by the primer-specific annealing temperature for 1 min; 95 °C for 1 min followed by 35 s at the annealing temperature; and a final step at 95 °C for 15 s. All reactions were performed in technical duplicates. Gene expression was normalized using the geometric mean of two housekeeping genes, *ef1a* and *40s*, validated for stability under the experimental conditions. Relative expression was calculated using the 2^–ΔΔCt^ method (28), and results were expressed as fold change relative to the control group.

**Table 2:** Sequences of the primer pairs used in the qPCR.

Gene	Sequence 5′→;3′	Annealing temperature (°C) intestine	Annealing temperature (°C) skin	Efficiency (%) intestine	Efficiency (%) skin	Slope intestine		Slope skin		Product size (bp)	Accession number
40s Ribosomal Protein (*40s*)	F - TGATTGTGACAGACCCTCGTG R - CACAGAGCAATGGTGGGGAT	60	60	102.91	104.59		-3.25		-3.22	79	HE978789.1
Elongation-factor 1α (*ef1a*)	F - AACTTCAACGCCCAGGTC AT R - CTTCTTGCCAGAACGACG GT	60	60	113.74	99.56		-3.03		-3.33	144	AJ866727.1
Arginase 2 (*arg2*)	F - TTGGCGACCTCAACTTCCAC R - CCCAGCATGACAAGGGTGTG	60	55	105.27	107.09		-3.20		-3.16	145	KM225768.1
Caspase 3 (*casp3*)	F - TGATGTCGTCTCTGCCGTAG R - ACCACCTCATACGCATCCTC	60	60	118.65	128.80		-2.94		-2.78	76	DQ345773
C-X-C Motif Chemokine Receptor 4 (*cxcr4*)	F - ACCAGACCTTGTGTTTGCCA R - ATGAAGCCCACCAGGATGTG	60	60	102.28	102.28		-3.27		-3.27	171	FN687464.1
Hepcidin Antimicrobial Peptide (*hamp*)	F - ACACTCGTGCTCGCCTTTAT R - TGTGATTTGGCATCATCCACG	57	57	113.53	116.16		-3.04		-2.99	148	KJ890396.1
Interleukin 10 (*il10*)	F - ACCCCGTTCGCTTGCCA R - CATCTGGTGACATCACTC	60	60	91.85	95.32		-3.53		-3.44	164	AM268529.1
Interleukin 1 Beta (*il1b*)	F - AGCGACATGGTGCGATTTCT R - CTCCTCTGCTGTGCTGATGT	60	60	86.94	127.91		-3.68		-2.80	105	AJ269472.1
Interleukin 8 (*il8*)	F - CGCTGCATCCAAACAGAGAGCAAAC R - TCGGGGTCCAGGCAAACCTCTT	60	60	117.29	109.24		-2.97		-3.12	140	AM490063.1
Macrophage Colony-Stimulating Factor Receptor 1 (*mscfr1*)	F - ATGTCCCAACCAGACTTTGC R - GGCTCATCACACACTTCACC	60	60	84.74	99.88		-3.75		-3.32	200	DLAgn_00109630
Macrophage Migration Inhibitory Factor (*mif*)	F - GCTCCCTCCACAGTATTGGCAAGAT R - TTGAGCAGTCCACACAGGAGTTTAGAGT	60	60	102.44	102.44		-3.26		-3.26	76	FN582353
Matrix Metallopeptidase 9 (*mmp9*)	F - TGTGCCACCACAGACAACTT R - TTCCATCTCCACGTCCCTCA	60	60	108.57	94.68		-3.13		-3.46	166	FN908863.1
Spermidine/Spermine N1-acetyltransferase (*sat*)	F - GCATCATCGCGAAATCCAAGGAGAGAACA R - CCAACCACCTTCAGGCCGTCACT	60	60	101.37	101.90		-3.29		-3.28	55	KM225772
Transforming Growth Factor Beta (*tgfb*)	F - ACCTACATCTGGAACGCTGA R - TGTTGCCTGCCCACATAGTAG	55	55	113.28	136.71		-3.04		-2.67	143	AM421619.1
Toll-like Receptor 2 (*tlr2*)	F - CAGTAGGCCAAGTCCGTCTC R - GGAGCTACGCTTGGCCTTTA	60	60	108.11	100.67		-3.14		-3.31	173	KX399288.1
Tumor Necrosis Factor Alpha (*tnfa*)	F - AGCCACAGGATCTGGAGCTA R - GTCCGCTTCTGTAGCTGTCC	55	55	116.52	127.65		-2.98		-2.80	112	DQ070246.1

### 2.6 Data analysis

The IBM SPSS Statistics for Windows was used to test data for normality and homogeneity of variance through the Shapiro-Wilk and Levene’s tests, respectively. Whenever necessary, outliers were removed, and data expressed as percentages were arcsine transformed (29) previous to analysis. Significant differences were determined by one-way analysis of variance (one-way ANOVA) followed by Tukey´s *post hoc* test. For all the tests, 95% CI was used, giving a probability level of 0.05. Data are presented as means ± standard error of the mean (SEM).

A multivariate canonical discriminant analysis (DA) was performed in XLSTAT v2023.1.2 (Addinsoft, New York, USA) using the gene-expression data from challenged European seabass sampled at 7 days post-infection, including biomarkers measured in the skin and intestine, to assess whether multigene expression profiles discriminated among dietary treatments. This approach identifies linear combinations of the original variables that maximize separation between predefined groups. Prior to analysis, data were checked for compliance with the assumptions of multivariate analysis. Overall model significance was assessed using Wilks’ lambda, whereas distances between group centroids were estimated using Mahalanobis distances. Pairwise group discrimination was further evaluated using Fisher’s distances and the corresponding p-values. The complete pairwise distance matrix is provided in the **Supplementary File**.

## 3 Results

Growth performance and feed utilization did not differ significantly among dietary treatments at the end of the 4 weeks of feeding, with no effects being observed on final body weight, weight gain or feed conversion ratio (**Table 3**). Moreover, cumulative mortality remained low (≤ 5.3%) and did not differ among dietary treatments (data not shown). Only a small proportion of infected fish exhibited mild external signs consistent with tenacibaculosis symptomatology, including localized skin erosion, with no severe ulcerative lesions being detected.

**Table 3:** Key performance indicators of European seabass fed a commercial-like diet (Control) and two diets supplemented with β-glucans (BG) at two different levels for four weeks.

Key performance indicator	Control	BG0.06	BG0.12	P-value
Initial weight (g)	11.32 ± 0.37	11.31 ± 0.36	11.32 ± 0.36	0.829
Final weight (g)	18.65 ± 0.80	18.89 ± 0.66	20.20 ± 0.44	0.219
Weight gain (%)	64.71 ± 7.05	66.89 ± 5.83	78.46 ± 3.93	0.322
FCR	1.43 ± 0.12	1.33 ± 0.10	1.26 ± 0.06	0.525
Survival (%)	95.00 ± 2.31	95.63 ± 1.75	91.25 ± 1.57	0.120

Values are presented as means ± SEM (n = 8). FCR, feed conversion ratio.

**Figure 2:**
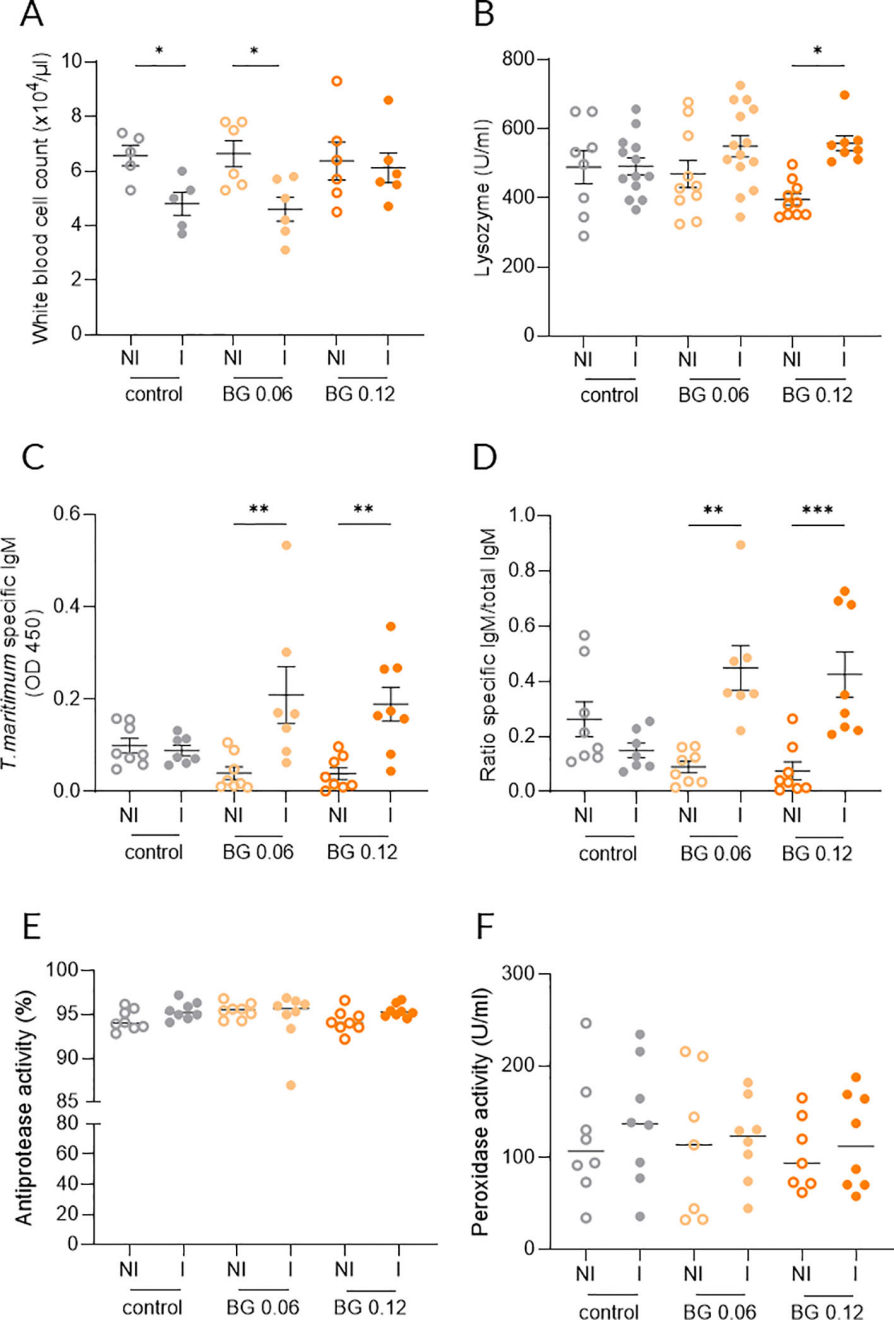
Innate and acquired immune responses of European seabass fed β-glucan (BG) supplemented diets at 24 h (and at 3 weeks only for total and specific IgM) after *T. maritium* bath challenge. **(A)** Circulating total white blood cells (n = 12); **(B)** Plasma lysozyme activity (n = 16); **(C)** Plasma specific IgM levels against *T. maritium* (n = 16); **(D)** Ratio of specific and total IgM in plasma (n = 16); **(E)** Plasma antiprotease activity (n = 12); **(F)** Plasma peroxidase activity (n = 12); NI: not infected, I: infected. Data are shown as means ± SEM. * = p < 0.05, ** = p < 0.01, *** = p < 0.001.

Peripheral white blood cell counts at 24 h post-infection decreased significantly in infected (I) European seabass compared to their non-infected (NI) counterparts in both Control and BG0.06 groups at 24 h post-infection, whereas no significant differences were detected in the BG0.12 group (**Figure 2A**). No significant differences were found regarding hemoglobin or red blood cell counts (**Supplementary Table 1**). While plasma lysozyme activity did not differ between NI and I seabass from the Control and BG0.06 dietary treatments at 24 h post-infection, a significant increase was detected in infected fish fed the BG0.12 diet (**Figure 2B**). *T. maritimum*-specific IgM levels at 3 weeks post-infection increased significantly in I European seabass compared to NI counterparts being fed both BG supplemented diets, whereas no significant changes were observed between NI and I seabass fed the Control diet (**Figure 2C**). The ratio of *T. maritimum*-specific IgM to total IgM followed the same trend with a significant increase observed in I European seabass compared to NI fish from both BG0.06 and BG0.12 dietary treatments (**Figure 2D**). At 24 h post-infection, no significant differences were observed in plasma antiproteases (**Figure 2E**) or peroxidase (**Figure 2F**) activities between I and NI European seabass across dietary treatments.

**Figure 3:**
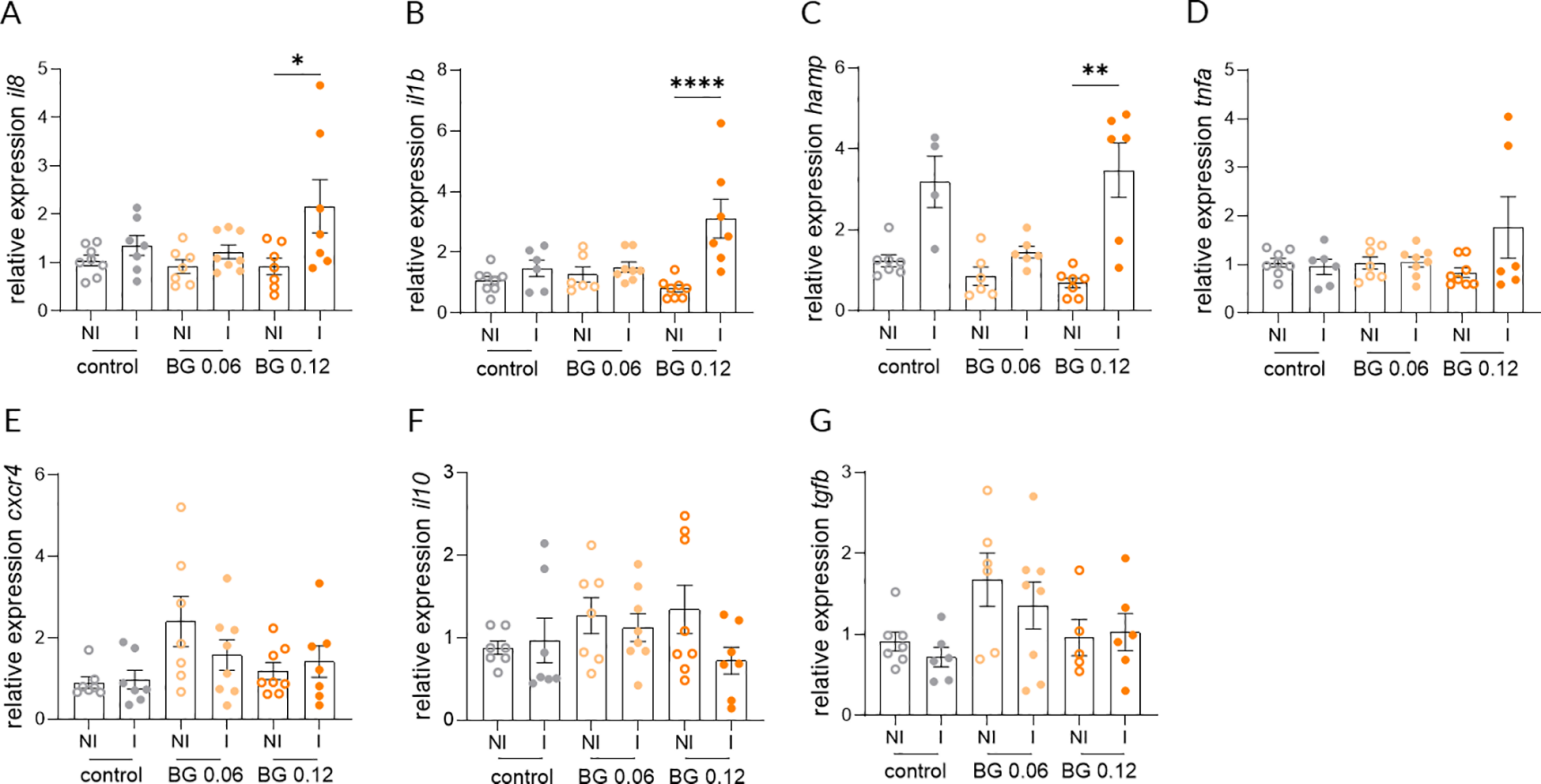
Relative gene expression in the skin of European seabass fed β-glucan (BG) supplemented diets at 24 h after *T. maritimum* bath challenge. **(A)** Interleukin 8; **(B)** Interleukin 1 beta; **(C)** Hepcidin antimicrobial peptide; **(D)** Tumor necrosis factor alpha; **(E)** c-x-c motif chemokine receptor 4; **(F)** Interleukin 10; **(G)** Transforming growth factor beta NI: not infected, I: infected. Data are shown as means ± SEM (n = 16). * = p < 0.05, ** = p < 0.01, **** = p < 0.0001.

**Figure 4:**
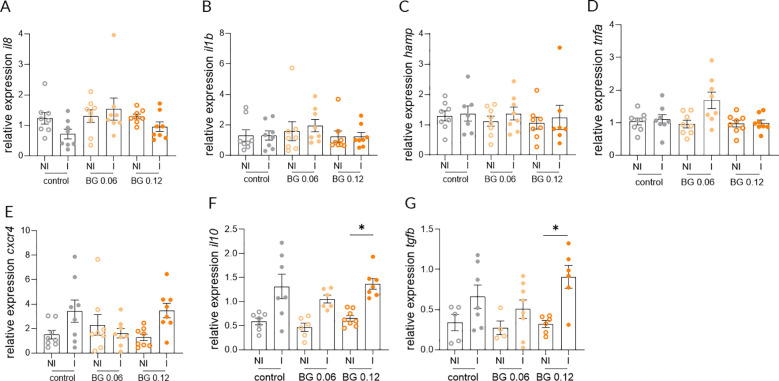
Relative gene expression in the skin of European seabass fed β-glucan (BG) supplemented diets at 7 days after *T. maritimum* bath challenge. **(A)** Interleukin 8; **(B)** Interleukin 1 beta; **(C)** Hepcidin antimicrobial peptide; **(D)** Tumor necrosis factor alpha; **(E)** c-x-c motif chemokine receptor 4; **(F)** Interleukin 10; **(G)** Transforming growth factor beta. NI: not infected, I: infected. Data are shown as means ± SEM (n = 16). * = p < 0.05.

**Figure 5:**
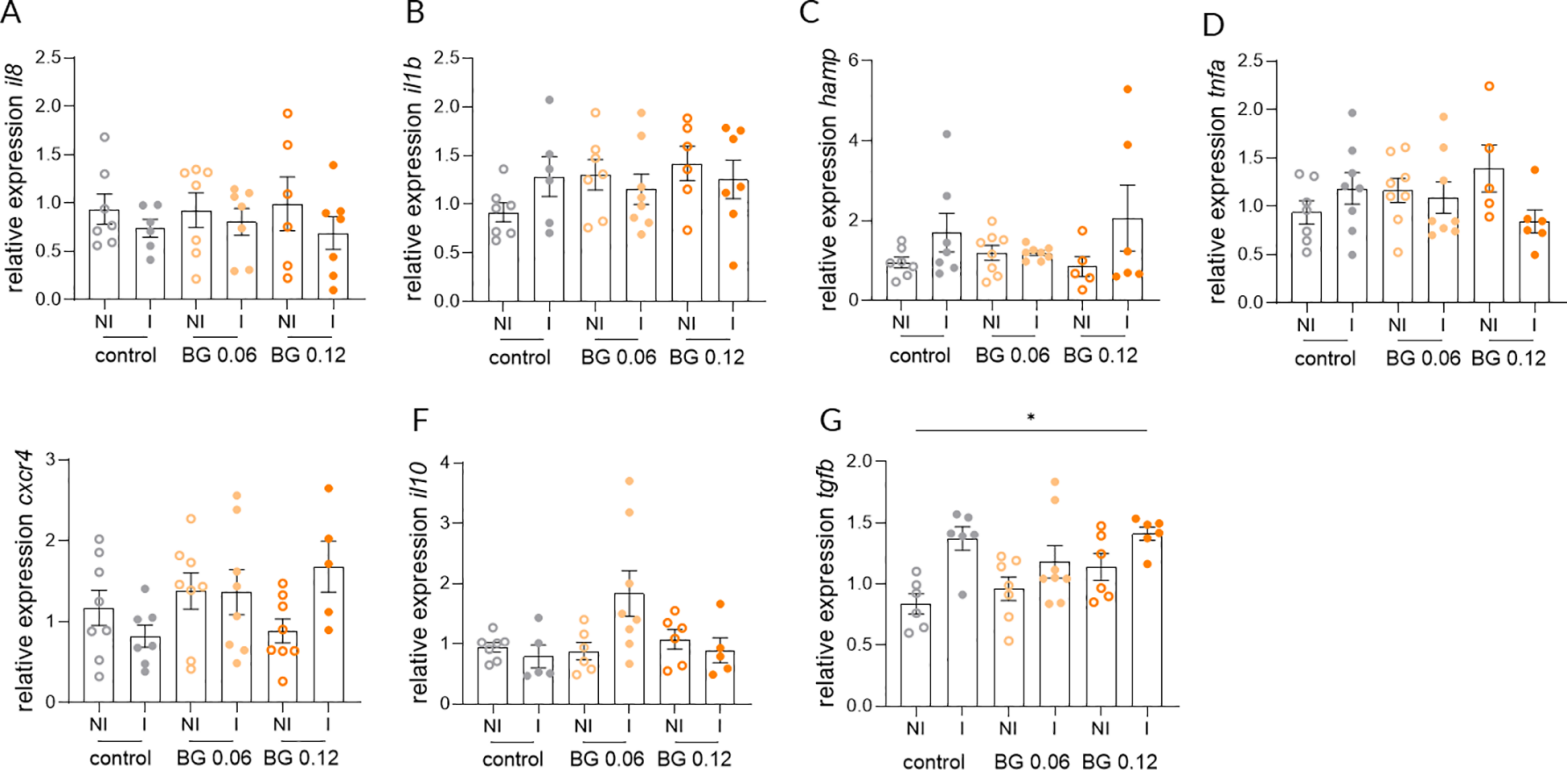
Relative gene expression in the intestine of European seabass fed β-glucan (BG) supplemented diets at 24 h after *T. maritimum* bath challenge. **(A)** Interleukin 8; **(B)** Interleukin 1 beta; **(C)** Hepcidin antimicrobial peptide; **(D)** Tumor necrosis factor alpha; **(E)** c-x-c motif chemokine receptor 4; **(F)** Interleukin 10; **(G)** Transforming growth factor beta. NI: not infected, I: infected. Data are shown as means ± SEM (n = 16). * = p < 0.05.

**Figure 6:**
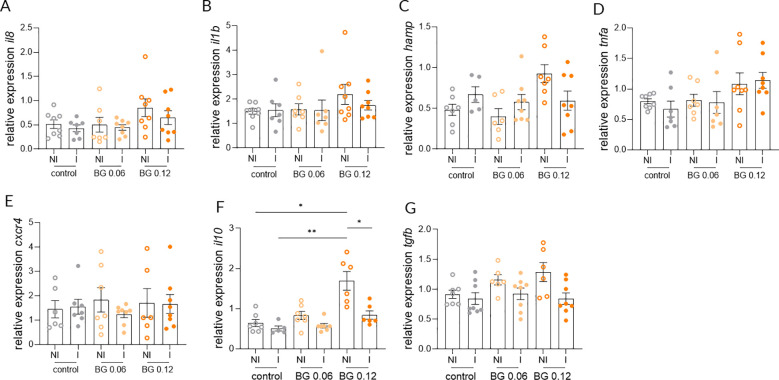
Relative gene expression in the intestine of European seabass fed β-glucan (BG) supplemented diets at 7 days after *T. maritimum* bath challenge. **(A)** Interleukin 8; **(B)** Interleukin 1 beta; **(C)** Hepcidin antimicrobial peptide; **(D)** Tumor necrosis factor alpha; **(E)** c-x-c motif chemokine receptor 4; **(F)** Interleukin 10; **(G)** Transforming growth factor beta. NI: not infected, I: infected. Data are shown as means ± SEM (n = 16). * = p < 0.05, ** = p < 0.01.

Relative expression of *il8*, *il1b* and *hamp* in the skin at 24 h post-infection did not differ significantly between NI and I European seabass fed the Control and BG0.06 diets, whereas a significant upregulation was detected in infected fish fed the BG0.12 diet (**Figures 3A–C**). In contrast the expression of *tnfa* and *cxcr4* in the skin did not differ significantly between NI and I fish in any of the groups at 24 h post-infection (**Figures 3D, E**). Similarly, no significant differences were detected in the expression of *il10* or *tgfb* in the skin across dietary treatments when comparing I and NI groups (**Figures 3F, G**). At 7 days post-infection, no significant differences in the expression of *il8*, *il1b*, *hamp*, *tnfa* and *cxcr4* were found in the skin, with transcript levels remaining comparable across all dietary groups (**Figures 4A–E**). However, significant upregulation of *il10* and *tgfb* expression was observed in the skin of I European seabass compared to their NI counterparts in the BG0.12 group, whereas no significant differences were detected in the Control or BG0.06 groups (**Figures 4F, G**). Additionally, no significant changes were detected for the remaining genes (*arg2*, *casp3*, *mcsfr1*, *mif*, *mmp9*, *sat* and *tlr2*) analyzed in the fish skin, whose expression remained stable across treatments at both sampling points (**Supplementary Table 2**). In the intestine, at 24 h post-infection, the expression of *il8*, *il1b*, *hamp*, *tnfa*, and *cxcr4* did not differ significantly between I and NI European seabass across dietary treatments (**Figures 5A–E**). Likewise, intestinal expression of *il10* did not differ significantly between NI and I European seabass across dietary treatments, whereas *tgfb* differed significantly only between NI European seabass fed the Control diet and I European seabass fed the BG0.12 diet (**Figures 5F, G**). At 7 days post-infection, intestinal expression of *il8*, *il1b*, *hamp*, *tnfa* and *cxcr4* remained comparable across all dietary groups (**Figures 6A–E**). In contrast, intestinal expression of *il10* differed significantly among dietary treatments (**Figure 6F**). Regarding NI and I European seabass fed with the Control diet, intestinal *il10* expression was significantly lower compared to NI fish fed the BG0.12 diet. Furthermore, within fish fed the BG0.12 diet, a significant downregulation in *il10* expression was observed in I European seabass compared to their NI counterparts. The expression of *tgfb* in the intestine did not differ significantly between I and NI European seabass in any of the dietary treatments at this time point (**Figure 6G**). The remaining intestinal markers (*arg2*, *casp3*, *mcsfr1*, *mif*, *mmp9*, *sat* and *tlr2*) also showed no significant modulation at either 24 h or 7 days post infection (**Supplementary Table 3**).

**Figure 7:**
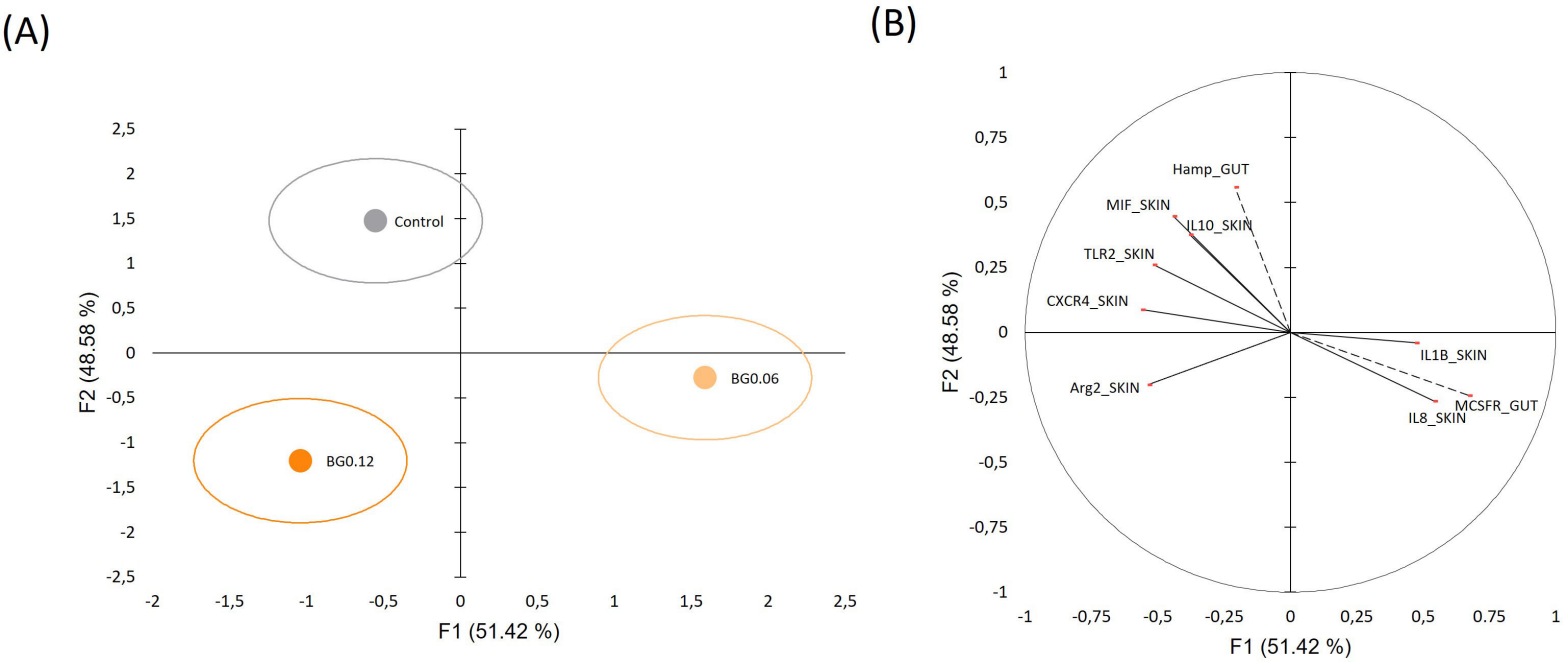
Canonical discriminant analysis of gene expression biomarkers in challenged European seabass at 7 days post-infection with **(T)** maritimum. **(A)** Canonical scores plot showing the distribution of dietary treatments (Control, BG0.06 and BG0.12). Small dots represent group centroids, whereas ellipses indicate data dispersion within each treatment group. **(B)** Loading plot showing the contribution of each biomarker to the two main discriminant functions (F1 and F2). Solid vectors represent skin biomarkers, whereas dashed vectors represent intestinal biomarkers.

To assess whether challenged fish displayed diet-dependent multigene expression patterns at 7 days post-infection, a DA was performed using selected skin and intestinal biomarkers. The overall model was significant (Wilks’ λ = 0.167; Rao’s F = 2.093; p = 0.042), and the first two canonical functions explained 51.42% and 48.58% of the total discriminatory variation, respectively. Group centroids indicated partial separation among CTRL, BG0.06 and BG0.12 challenged fish (**Figure 7A**). However, pairwise Fisher distances were not significant (p = 0.103–0.118), indicating that the multivariate response should be interpreted as an overall dietary structuring rather than as significant pairwise discrimination between diets. The variables contributing most strongly to group separation were predominantly skin biomarkers, whereas the contribution of intestinal markers was more limited (**Figure 7B**).

## 4 Discussion

The increasing incidence of bacterial diseases such as tenacibaculosis remains a major challenge for marine aquaculture, given the pathogens ability to colonize mucosal tissues and rapidly induce lesions (8), this highlights the needs of preventive approaches that reinforce host defenses without compromising growth or welfare (8, 10). Among the different nutritional strategies proposed, yeast-derived BG have been widely investigated and applied as feed additives due to their capacity to modulate innate immune signaling, enhance microbial clearance and support mucosal health in teleost fish (3, 30–33). The present study evaluated two dietary inclusion levels of yeast-derived dietary BG in European seabass and assessed their immunomodulatory effects following an experimental *T. maritimum* challenge.

Although the primary aim of this study was to evaluate immune and inflammatory responses, growth performance and feed efficiency were also monitored to ensure that the tested inclusion levels did not compromise fish condition and zootechnical performance. Growth performance and FCR were not affected by dietary BG supplementation, indicating that both inclusion levels (0.06 and 0.12%) were well tolerated by European seabass after 4 weeks of feeding. Consistent with results from the present study, Zhang et al. (34) reported no impact of dietary BG supplementation on growth or feed conversion ratio in largemouth bass (*Micropterus salmoides*) following eight weeks of feeding at an inclusion level of 0.1%, despite measurable immune benefits. In a study with Nile tilapia (*Oreochromis niloticus*) fed 0.1-0.2% BG for 60 days, Barros et al. (35) reported no effects on growth performance nor feed efficiency, even when immunological parameters were modulated. Therefore, available data from literature together with results from the present study, reinforce that practical dietary BG inclusion levels can be adopted without compromising fish condition while benefiting immune responses.

In the present study, dietary BG supplementation modulated systemic immune readiness following *T. maritimum* infection. Circulating total WBCs constitute a primary defense layer, including phagocytes, antigen presenting cells and cytokine secretion during infection (36). At 24 h following bacterial challenge, infected fish fed control and BG0.06 diets exhibited a marked reduction in circulating WBC counts, consistent with an acute infection-induced leukopenia, similarly to that observed by Ferreira et al. (20) in European seabass bath-challenged with *T. maritimum*. In contrast, WBC counts in infected fish fed the BG0.12 diet remained stable, indicating a preserved immune homeostasis and suggesting enhanced resilience against acute leukocyte depletion. This pattern aligns with previous observations in other teleosts, where dietary BG mitigated stress or infection-associated leukocyte drops. In Nile tilapia, BG supplementation prevented acute stress-induced leukopenia (37) and stabilized leukocyte dynamics during bacterial challenge (35, 38). Similarly, BG feeding maintained leukocyte profiles while modulating cortisol response in other fish species (39, 40), supporting the role of BG in preserving immune competence under acute stress. In addition to conserving circulating leukocyte availability, BG have been described to enhance the functional capacity of innate immune cells. In a study with gilthead seabream, dietary *S. cerevisiae* BG promoted increased phagocytic activity, respiratory burst and plasma myeloperoxidase levels in circulating leukocytes (41). Therefore, these previous findings complement hematological observations from this study, and it can be suggested that dietary BG supplementation not only preserves circulating leukocyte counts but also improves their functional competence during infection.

Lysozyme is a key enzyme that can be produced by innate immune cells such as neutrophils and macrophages, contributes to bacterial cell wall lysis and constitutes a critical early barrier against infection since it is present in mucosal surfaces (42). In the present study, fish fed the BG0.12 diet showed significantly higher plasma lysozyme activity following *T. maritimum* infection, indicating that supplementation at this level enhanced rapid humoral responses during the acute phase of disease. Similar effects have been observed in other teleosts, where dietary inclusion of 0.1% BG enhanced lysozyme responses under bacterial challenge conditions as reported in pacu (*Piaractus mesopotamicus*) following exposure to heat-killed *Aeromonas hydrophila* (40), and in largemouth bass during *A. schubertii* infection, where increased lysozyme activity coincided with improved survival rate (34). These consistent findings align with recent reports indicating that dietary BG can enhance effector mechanisms, including lysozyme activity, thereby supporting disease resistance in aquaculture species (33). Taken together, the increased lysozyme activity observed in the present study, supports the role of BG in reinforcing early antibacterial defenses during a bacterial challenge.

Specific and total IgM are critical components of adaptive immunity in fish and play a central role in the initial humoral response to pathogens (43). In the current study, specific IgM against *T. maritimum* increased significantly in I fish fed BG-supplemented diets, while the levels remained unchanged in the control groups at 3 weeks after infection. The ratio between specific and total IgM followed the same pattern, indicating a targeted response driven by dietary BG rather than a generalized immunoglobulin increase after infection. These data suggest an earlier immunization state in infected fish fed BG compared to those fed the control diet, and the potential synergistic effect of dietary BG and vaccination strategies should be further explored in future studies. Direct evidence supporting BG modulation of IgM and B-cell responses in teleosts has recently emerged. Martín et al. (44) fed rainbow trout a BG-supplemented diet (0.1% supplementation) for one month and demonstrated enhanced IgM^+^ B-cell and IgM-secreting capacity, together with improved antigen processing and phagocytic abilities in circulating B-cells. Although B-cell populations were not evaluated in the present study, these findings provide relevant mechanistic support, showing that dietary BG can favor more efficient IgM-mediated responses following infection.

Skin is among the primary barriers to be affected by *T. maritimum*, where epithelial integrity and local chemokine/cytokine balance dictate early containment of the pathogen (5). In the present study, dietary BG supplementation, particularly at the highest level, promoted a pronounced and targeted early transcriptional activation in skin tissue 24 h post-infection, characterized by a significant upregulation of *il8*, *il1b*, and *hamp*, while *tnfa* and *cxcr4* remained unchanged. While this response profile reflects a rapid but spatially confined inflammatory activation in the infected skin, it could also suggest a trained immunity signature driven by activated European seabass phagocytes. The coordinated induction of *il8* and *il1b* indicates a robust chemotactic and pro-inflammatory signaling cascade favoring leukocyte recruitment, while the increase in *hamp* supports the activation of antimicrobial defenses based on restricted iron availability. The absence of *tnfa* modulation may suggest that BG supplementation favored an effective yet controlled inflammatory reaction, preventing tissue damage while maintaining immune efficacy. Evidence from other teleost species supports this interpretation. In common carp (*Cyprinus carpio*), BG administration prior to *Aeromonas hydrophila* infection enhanced phagocytic activity, increased leukocyte counts and improved survival, despite stable *il1b* levels (45). This indicates that BG potentiates innate effector functions rather than sustaining excessive cytokine expression, consistent with the efficient but contained response in this study. Comparable activation of *il10* and *il8* has also been demonstrated in rainbow trout following BG feeding during *Aeromonas hydrophila* challenge (46) and in Nile tilapia infected with *Streptococcus iniae* (47), highlighting the conserved nature of this early inflammatory response across teleosts. The character of this response aligns with previous observations, where BG stimulation via toll-like and dectin-1-like receptors induced a balanced pro- and anti-inflammatory response (48). Such receptor-mediated modulation may explain the absence of *tnfa* upregulation in the present study, indicating that BG promote early defense activation while preventing uncontrolled inflammation in the tissue. Although additional genes involved in macrophage signaling and tissue remodeling (*mmp9*, *tlr2*, *mif*, *arg2*) were analyzed, their expression was not significantly altered, suggesting that the transcriptional response at this stage of infection was primarily focused on chemotaxis and antimicrobial activity. By 7 days post-infection, a clear regulatory shift was observed, particularly in fish fed with BG0.12, with a significant upregulation of *il10* and *tgfb*. These cytokines are central to the resolution of the inflammation phase, limiting tissue damage and promoting tissue repair. This coordinated increase of these anti-inflammatory mediators suggests BG supplementation favored an efficient transition from activation to regulation, restoring epithelial homeostasis after the acute phase of *T. maritimum* infection. Similar immune kinetics were observed in common carp fed a 0.1% BG-supplemented diet for 14 days before a challenge with *Aeromonas hydrophila* (45). In that study, the authors observed early stimulation of macrophage phagocytic activity followed by an increase in *il10* expression, a pattern like the one we obtained in the present work, demonstrating that BG can induce a time-dependent switch from a pro-inflammatory to a regulatory state, enabling efficient pathogenic clearance without chronic activation. In Atlantic salmon, Rodríguez et al. (49) reported that fish fed with BG-supplemented diets (0.1%, 4 weeks) showed enhanced *il10* transcription in head-kidney after vaccination and confinement stress. The authors interpreted this upregulation as part of the adaptive feedback loop preventing overstimulation of immune pathways under prolonged antigenic exposure, aligning with the results of the present study where increased *il10* and *tgfb* expression at 7 days post-infection likely reflects a feedback mechanism, supporting immune resolution and tissue repair in the infected skin. Taken together, the increase of *il10*, and *tgfb* at 7 days post-infection, following the early activation of *il8*, *il1b* and *hamp* at 24 h post-infection suggests that BG supplementation can promote a dynamic and self-limited immune response in European seabass. This sequence, from pro-inflammatory activation to anti-inflammatory regulation, supports infection control, minimizes tissue damage and may facilitate skin regeneration after the ulcerative lesions caused by *T. maritimum*. Consistent with this interpretation, the DA supported the univariate qPCR data by showing that dietary treatments differed more clearly when biomarkers were considered jointly compared to each transcript assessed individually. Importantly, the markers contributing most to group discrimination were mainly from the skin, reinforcing the view that response to *T. maritimum* was centered on the primary mucosal interface. Nevertheless, because pairwise Fisher distances were not significant, this multivariate analysis should be interpreted cautiously and viewed as evidence of an overall coordinated dietary effect rather than definitive separation between individual diets. Although β-glucan-induced long-term modulation of innate immune function has been documented in systemic compartments of teleosts, particularly in macrophages and neutrophils, which can display enhanced responsiveness after stimulation (19, 50, 51), the present study does not allow conclusions regarding the existence of memory-like phenomena in innate immune cells from mucosal tissues such as skin. In mammals, β-glucans are well known to induce sustained metabolic end epigenetic remodeling in monocytes and macrophages, resulting in heightened antimicrobial performance (52, 53). However, whether similar durable reprogramming can occur in mucosal barriers of fish remains unclear. Given these considerations, the rapid but well-regulated cytokine response observed in the skin of BG-fed European seabass could be more appropriately interpreted as an enhanced local innate readiness, rather than evidence of persistent reprogramming. Dedicated experiments, such as withdrawal studies or secondary challenges, would be required to determine whether innate immune cells from mucosal surfaces can acquire long-lasting functional changes following β-glucan supplementation.

Regarding the intestinal response, it remained unaltered at 24 h post-infection, with no changes detected in *il8*, *il1b*, *hamp*, *tnfa*, or *cxcr4*, indicating that the immune activation induced by *T. maritimum* was restricted to the main site of infection. Rather than reflecting an absence of BG responsiveness, this pattern is consistent with a targeted local response centered in the skin, the primary portal of this pathogen entry and the most affected tissue during tenacibaculosis. A similar compartmentalized response has been observed previously in European seabass, where transcriptional activation was confined to the site of contact, while distal mucosal tissues remained stable despite a systemic immune stimulation (20). At 7 days post-infection, intestinal *il10* expression differed significantly among dietary treatments, with European seabass fed the BG0.12 diet displaying higher basal (NI) transcriptional levels compared to fish fed the Control of BG0.06 diets. Notably, infection-associated differences were only detected within the fish fed with the BG0.12 diet, where *il10* expression was significantly reduced in I fish relative to their NI counterparts. This response pattern contrasts with the regulatory shift observed in skin, where *il10* and *tgfb* were selectively upregulated in I fish fed the BG0.12, and indicates that intestinal immune regulation follows a distinct trajectory. Rather than transitioning toward an anti-inflammatory state during infection, the intestine appears to restrain a more regulatory signaling in BG0.12 fed fish, possibly maintaining immune alertness during the resolution phase occurring at the primary site of infection. Such compartmentalized immune regulation is consistent with previous reports showing that dietary BG do not induce an uniform activation across tissues, but instead modulate immune responsiveness in a context and tissue dependent manner. In Atlantic salmon, Rodríguez et al. (49) reported that BG feeding elicited subtle effects under basal conditions, whereas stronger immune modulation occurred when supplementation was combined with vaccination. Although intestinal responses were not assessed in that study, their findings support the concept thar BG-induced priming enhances responsiveness primarily at sites of immunological challenge. In line with this, results from this study suggest that BG supplementation promotes localized regulatory resolution in the skin while maintaining a more controlled regulatory profile in the intestine, thereby avoiding unnecessary immunosuppression during recovery.

Taken together, the present findings reveal a consistent pattern of BG-induced immune modulation in European seabass, characterized by an early inflammatory and antimicrobial activation followed by a regulated resolution phase. The increase in leukocyte counts and lysozyme activity, together with an early up-regulation of *il8*, *il1b* and *hamp* in the skin, indicates that dietary BGs accelerate the initial host response at the infection site. This tissue-specific response is further supported by the multivariate analysis, where the variables contributing most strongly to group discrimination were predominantly skin biomarkers, highlighting the central role of the skin as the primary responsive interface during *T. maritimum* infection. The later induction of *il10* and *tgfb* suggests an effective regulatory shift that limits inflammation and promotes tissue recovery. This pattern illustrates a balanced innate immune response to regulatory transition that favors pathogen control while minimizing tissue damage. From a broader perspective, these results also confirm that the verified BG-induced immunomodulation can be achieved without compromising growth performance. The stronger responses observed at the higher inclusion level (0.12%) reinforce the importance of dietary dose in achieving immunological benefits and provide practical guidance for functional feed formulation in aquaculture.

In conclusion, dietary BGs supplementation enhanced the capacity of European seabass to respond to *T. maritimum* under the present experimental conditions, promoting a coordinated immune response characterized by early innate activation, controlled inflammatory regulation and increased production of *T. maritimum*-specific IgM. Notably, these effects were most consistent at the higher inclusion level tested (0.12%), highlighting this dosage as a promising functional level for immune modulation in this species. Rather than inducing generalized immune activation, BG supplementation at 0.12% modulated immune responsiveness in a tissue-specific manner, enhancing early defense and subsequent regulatory mechanisms at the primary site of infection. Collectively, these findings provide a strong proof of concept that dietary BGs can enhance mucosal and systemic immune readiness against opportunistic pathogens such as *T. maritimum*. Future studies should focus on determining the persistence of BG-induced immune priming after dietary withdrawal and assessing whether short-term supplementation can provide lasting protection and evaluating possible synergies with vaccination strategies. In addition, validation under commercial farming conditions will be essential to determine the translational applicability and long-term benefits of this nutritional approach. Given that *T. maritimum* infection causes ulcerative skin lesions, it would also be relevant to investigate whether BG supplementation supports skin repair and regeneration during disease recovery.

## Data Availability

The data presented in the study are deposited in the “Figshare” repository: https://doi.org/10.6084/m9.figshare.31852756.
