# Supplementary material for: Dietary 1.3-1.6 yeast β-glucans enhance immune response and disease resilience in European seabass challenged with Tenacibaculum maritimum
Source: Front Immunol. 2026 Apr 20;17:1798375. doi: 10.3389/fimmu.2026.1798375 (PMC13136018; doi:10.3389/fimmu.2026.1798375)
Supplement: Supplementary file 1 [file DataSheet1.docx]

Supplementary Tables

Supplementary Table 1. Hematological parameters of European seabass 24 h after *T.maritimum* challenge. Data are shown as means ± SEM (n = 12). Results are not significantly different (p > 0.005).

| Hematological parameters | Non-Infected | | | Infected | | |
| --- | --- | --- | --- | --- | --- | --- |
|  | Control | BG0.06 | BG0.12 | Control | BG0.06 | BG0.12 |
| Hemoglobin (g dL^-1^) | 3.15 ± 0.16 | 3.07 ± 0.11 | 3.05 ± 0.17 | 3.06 ± 0.16 | 3.25 ± 0.19 | 3.81 ± 0.30 |
| Red blood cells (x10^6^ CFU mL^-1^) | 3.03 ± 0.18 | 2.73 ± 0.16 | 2.45 ± 0.13 | 2.66 ± 0.12 | 2.46 ± 0.13 | 3.16 ± 0.23 |

Supplementary Table 2. Relative expression of arginase 2 (*arg2*), caspase 3 (*casp3*), macrophage colony-stimulating factor receptor 1 (*mcsfr1*), macrophage migration inhibitory factor (*mif*), matrix metallopeptidase 9 (*mmp9*), spermidine/spermine n1-acetyltrasnferase (*sat*) and toll-like receptor 2 (*tlr2*) in the skin of European seabass fed β-glucan (BG) supplemented diets at 24 h and 7 days after *T. maritimum* challenge. Data are expressed as mean ± SEM (n = 8). Results are not significantly different (p > 0.005) at either 24 h or 7 days post-infection.

| **Genes** | **Non-Infected** | | | | | | **Infected** | | | | | |
| --- | --- | --- | --- | --- | --- | --- | --- | --- | --- | --- | --- | --- |
|  | **24 h post-infection** | | | **7 days post-infection** | | | **24 h post-infection** | | | **7 days post-infection** | | |
|  | **Control** | **BG0.06** | **BG0.12** | **Control** | **BG0.06** | **BG0.12** | **Control** | **BG0.06** | **BG0.12** | **Control** | **BG0.06** | **BG0.12** |
| ***arg2*** | 1.17 ± 0.24 | 072 ± 0.08 | 0.49 ± 0.07 | 0.75 ± 0.07 | 0.87 ± 0.09 | 0.67 ± 0.12 | 0.84 ± 0.14 | 0.73 ± 0.06 | 0.74 ± 0.08 | 0.89 ± 0.14 | 1.43 ± 0.26 | 1.34 ± 0.7 |
| ***casp3*** | 1.00 ± 0.02 | 0.97 ± 0.10 | 0.72 ± 0.05 | 0.48 ± 0.06 | 0.44 ± 0.06 | 0.42 ± 0.05 | 1.20 ± 0.17 | 0.94 ± 0.17 | 1.26 ± 0.25 | 0.64 ± 0.07 | 0.77 ± 0.13 | 0.70 ± 0.04 |
| ***mcsfr1*** | 1.02 ± 0.07 | 0.95 ± 0.09 | 0.80 ± 0.10 | 0.62 ± 0.07 | 0.81 ± 0.11 | 0.84 ± 0.12 | 1.52 ± 0.23 | 1.04 ± 0.11 | 1.39 ± 0.23 | 0.82 ± 0.09 | 1.00 ± 0.15 | 1.00 ± 0.06 |
| ***mif*** | 1.02 ± 0.07 | 0.90 ± 0.04 | 0.09 ± 0.07 | 1.02 ± 0.08 | 1.19 ± 0.16 | 1.12 ± 0.07 | 1.24 ± 0.12 | 1.00 ± 0.11 | 1.87 ± 0.49 | 1.37 ± 0.16 | 2.02 ± 0.44 | 1.11 ± 0.11 |
| ***mmp9*** | 1.10 ± 0.18 | 1.52 ± 0.18 | 1.28 ± 0.27 | 0.63 ± 0.09 | 0.71 ± 0.23 | 0.52 ± 0.07 | 2.02 ± 0.53 | 2.43 ± 0.38 | 2.21 ± 0.47 | 0.70 ± 0.14 | 1.64 ± 0.26 | 0.98 ± 0.14 |
| ***sat*** | 1.04 ± 0.10 | 1.18 ± 0.13 | 0.76 ± 0.10 | 0.82 ± 0.10 | 0.50 ± 0.06 | 0.51 ± 0.10 | 1.10 ± 0.14 | 0.95 ± 0.14 | 1.52 ± 0.39 | 0.52 ± 0.21 | 0.81 ± 0.11 | 0.66 ± 0.07 |
| ***tlr2*** | 1.07 ± 0.12 | 1.11 ± 0.12 | 0.81 ± 0.10 | 0.85 ± 0.09 | 0.73 ± 0.08 | 0.78 ± 0.08 | 1.55 ± 0.06 | 0.95 ± 0.11 | 1.46 ± 0.44 | 1.19 ± 0.11 | 0.99 ± 0.14 | 1.14 ± 0.10 |

Supplementary Table 3. Relative expression of arginase 2 (*arg2*), caspase 3 (*casp3*), macrophage colony-stimulating factor receptor 1 (*mcsfr1*), macrophage migration inhibitory factor (*mif*), matrix metallopeptidase 9 (*mmp9*), spermidine/spermine n1-acetyltrasnferase (*sat*) and toll-like receptor 2 (*tlr2*) in the intestine of European seabass fed β-glucan (BG) supplemented diets at 24 h and 7 days after *T. maritimum* challenge. Data are expressed as mean ± SEM (n = 8). Results are not significantly (p > 0.005) different at either 24 h or 7 days post-infection.

| **Genes** | **Non-Infected** | | | | | | **Infected** | | | | | |
| --- | --- | --- | --- | --- | --- | --- | --- | --- | --- | --- | --- | --- |
|  | **24 h post-infection** | | | **7 days post-infection** | | | **24 h post-infection** | | | **7 days post-infection** | | |
|  | **Control** | **BG0.06** | **BG0.12** | **Control** | **BG0.06** | **BG0.12** | **Control** | **BG0.06** | **BG0.12** | **Control** | **BG0.06** | **BG0.12** |
| ***arg2*** | 3.21 ± 1.64 | 3.44 ± 2.00 | 7.21 ± 4.99 | 3.53 ± 1.20 | 13.46 ± 6.96 | 3.88 ± 2.06 | 10.28 ± 3.84 | 2.17 ± 1.06 | 10.41 ± 4.35 | 2.38 ± 0.65 | 6.67 ± 2.38 | 6.95 ± 3.50 |
| ***casp3*** | 1.05 ± 0.12 | 1.05 ± 0.13 | 1.19 ± 0.20 | 0.83 ± 0.08 | 1.04 ± 0.26 | 0.88 ± 0.20 | 1.31 ± 0.43 | 1.19 ± 0.20 | 1.70 ± 0.09 | 0.63 ± 0.07 | 0.65 ± 0.06 | 0.63 ± 0.08 |
| ***mcsfr1*** | 1.08 ± 0.13 | 1.17 ± 0.16 | 1.30 ± 0.16 | 1.27 ± 0.12 | 1.25 ± 0.18 | 1.27 ± 0.19 | 1.36 ± 0.22 | 1.21 ± 0.10 | 1.08 ± 0.18 | 0.50 ± 0.12 | 0.94 ± 0.10 | 0.58 ± 0.10 |
| ***mif*** | 1.10 ± 0.18 | 1.60 ± 0.45 | 1.17 ± 0.21 | 1.37 ± 0.14 | 1.45 ± 0.05 | 1.61 ± 0.18 | 0.82 ± 0.10 | 0.90 ± 0.05 | 0.69 ± 0.06 | 1.29 ± 0.15 | 1.29 ± 0.21 | 1.25 ± 0.14 |
| ***mmp9*** | 1.75 ± 0.47 | 0.93 ± 0.40 | 1.83 ± 0.68 | 0.94 ± 0.41 | 0.26 ± 0.09 | 1.02 ± 0.33 | 1.13 ± 0.24 | 2.63 ± 0.49 | 1.35 ± 0.27 | 0.80 ± 0.32 | 0.48 ± 0.16 | 0.27 ± 0.09 |
| ***sat*** | 1.04 ± 0.11 | 1.18 ± 0.11 | 1.11 ± 0.13 | 0.90 ± 0.11 | 0.89 ± 0.11 | 0.97 ± 0.12 | 1.27 ± 0.21 | 1.21 ± 0.11 | 1.08 ± 0.13 | 0.78 ± 0.13 | 0.77 ± 0.13 | 0.75 ± 0.07 |
| ***tlr2*** | 1.27 ± 0.28 | 0.52 ± 0.07 | 0.45 ± 0.08 | 0.60 ± 0.18 | 0.48 ± 0.09 | 0.62 ± 0.17 | 0.76 ± 0.17 | 0.70 ± 0.14 | 0.60 ± 0.13 | 0.36 ± 0.08 | 0.36 ± 0.08 | 0.41 ± 0.07 |
